# Regime Shifts in Microbial and Water Quality Dynamics in Red Tilapia Ponds

**DOI:** 10.3390/microorganisms13071553

**Published:** 2025-07-02

**Authors:** Ziyan Liu, Jiaqi Li, Lei Luo, Yang Yu, Jianing Yan, Caiyun Sun, Xiangjun Miao, Wensheng Li

**Affiliations:** 1 State Key Laboratory of Biocontrol, Guangdong Province Key Laboratory for Aquatic Economic Animals, Guangdong Provincial Engineering Technology Research Center for Healthy Breeding of Important Economic Fish, School of Life Sciences, Sun Yat-Sen University, Guangzhou 510275, China; liuziyan95@163.com (Z.L.);; 2 Yunnan Academy of Fishery Sciences, Kunming 650224, China

**Keywords:** water quality indicators, microalgal-bacterial, aquaculture ponds, tilapia

## Abstract

Changes in the aquatic ecological environment have a significant impact on aquaculture efficiency. In order to understand the changes in water quality and the dynamics of microalgae and bacteria in the process of aquaculture, 16S rRNA and 18S rRNA high-throughput sequencing technologies were used to determine the microorganisms in a red tilapia (*Oreochromis* sp.) aquaculture pond. During the breeding period (from 6 July 2023 to 13 November 2023), water samples were collected from three ponds, on average once every 20 days. The results of water quality analysis showed that at the end of culture (13 November 2023), the concentrations of NH_4_^+^-N and NO_2_^−^-N increased significantly, and both the air temperature (36.00 ± 0.00 to 21 ± 0.00 °C) and water temperature (32.83 ± 0.29 to 22.75 ± 0.42 °C) decreased significantly. The NH_4_^+^-N and NO_2_^−^-N concentrations increased by 597% (0.67 ± 0.17 to 4.67 ± 0.33 mg/L) and 782% (0.34 ± 0.16 to 3.00 ± 1.15 mg/L), respectively, from T1 to T6. Bacterial diversity decreased to T3 and then increased. The relative abundance of *hgcI_clade* (from 14.91% to 7.18%) and *CL500-29_marine_group* (from 3.35% to 1.39%) in aquaculture water generally decreased with the extension of aquaculture time. The abundance of *Komma* increased from T1 (1.44%) to T3 (13.90%) and decreased from T3 to T6 (4.21%). The pH, dissolved oxygen concentration, and temperature were main factors affecting the dynamics of bacteria, while dissolved oxygen, NH_4_^+^-N, and NO_2_^−^-N concentrations affected that of microalgae. In conclusion, this study revealed regime shift in the water quality and microalgal–bacterial community with increasing culture time in red tilapia aquaculture ponds.

## 1. Introduction

The aquaculture industry has experienced rapid growth in recent years due to increasing demand for seafood and declining wild fish stocks [1]. However, this expansion has come with unintended consequences, including environmental pollution and the spread of aquatic diseases. In feed-driven aquaculture systems, approximately 75% of feed nitrogen (N) and phosphorus (P) are not utilized effectively and remain as aquaculture waste, resulting in environmental risks [2,3,4]. This can lead to eutrophication, where excess nutrients in the aquaculture water cause microalgal blooms and oxygen depletion, harming aquaculture species and disrupting the balance of the ecosystem. Intensive modeling, excess nutrients, and inferior diversity can weaken the immune systems of farmed animals, making them more susceptible to pathogens [5,6]. If not properly managed, these pathogens can escape into the surrounding environment, potentially causing widespread mortality and ecosystem disruption. Therefore, it is crucial to investigate the changes in the aquatic ecological environment during the culture process.

The aquatic ecological environment includes water quality and microalgal–bacterial dynamics. In aquaculture, water quality is key to maintaining the health and welfare of aquatic animals. Water quality not only directly affects the growth and health of cultured animals [7] but also profoundly influences the microbial communities within the water [8,9]. For instance, it has been reported that a high NH_4_^+^-N level in water could induce an imbalance of the internal environment, eventually resulting in the gill, kidney, and liver injury of Persian sturgeon (*Acipenser persicus*) and stellate (*Acipenser stellatus*) [10]. High pH value (9.0) could suppress growth performance, induce oxidative stress, and inflammation of Chinese mitten crab (*Eriocheir sinensis*) [11]. The survival of Atlantic bluefin tuna (*Thunnus thynnus*) larvae was affected by environmental salinity rather than environmental pH from 7.3 to 8.0 [12]. Interestingly, the aquaculture water microbiome might be served as a keystone for understanding the disease etiology [13], since there is often no significant difference in water quality indicators of healthy and unhealthy aquaculture ponds in actual breeding [14]. There was a close relationship between the dramatic changes in bacterioplankton composition and the mass mortality of shrimp, implying that the compositional shifts in the microbiota could indicate the health status of shrimp in culture ponds [15]. A previous study on European perch (*Perca fluviatilis*) found that the bacterial community was linked with several water quality indicators, including temperature, salinity, and dissolved oxygen [16]. Many microorganisms, such as *Flavobacteria* and *Sphingobacteria*, can fortify the mineralization and decomposition of residual feeds and feces to improve water quality [17,18]. It has been reported that napA-harboring *Bradyrhizobium* spp. increased under higher levels of N fertilization, indicating that *Bradyrhizobium* spp. is a key player in nitrogen cycling processes [19]. For example, microalgae can transform inorganic nitrogen pollutants in water into biomass through photosynthesis while generating oxygen [20]. Similarly, bacteria can incorporate inorganic nitrogen pollutants into their biomass or convert them into less harmful nitrogen-containing compounds under specific conditions [21], facilitating the removal of nitrogen-containing pollutants. Moreover, microorganisms serve as a valuable food source for aquaculture species, such as algae (Bacillariophyta) and zooplankton (Rotifer), thereby reducing the need for external feed [22]. In addition, microorganisms can establish beneficial bacterial properties that compete with pathogens and reduce the morbidity of aquaculture species, such as bacteria of the genus *Bacillus* spp. [23]. Given the important role of microorganisms in the aquatic environment, they have been widely used in municipal wastewater treatment and aquaculture practices [24,25,26]. However, the changes in environmental factors, their interaction with the microbial community, and the fraction of pathogenic bacteria are not well-known in aquaculture systems. Understanding the structure and diversity of the microbial community present in aquaculture systems and their connection with the surrounding environment is of great importance in controlling the occurrence of aquaculture diseases.

Tilapia is the most widely cultivable fish group globally because of its high growth rate and good adaptability [1]. Among the different tilapia species currently farmed, the hybrid red tilapia (*Oreochromis* sp.) is important for commercial aquaculture since it possesses superior morphological characteristics (shape and color) and tolerance to environmental stress, such as salinity and pH [27,28]. In addition, red tilapia is an important tilapia farming species in Guangzhou, China. Thus, a comprehensive analysis of water quality and microbial dynamics in red tilapia ponds should be considered for evaluating the aquatic ecological environment. Regime shifts are large, abrupt, and persistent critical transitions in the function and structure of ecosystems [29,30]. However, it is unknown how these transitions will interact or whether the occurrence of one will increase the likelihood of another or simply correlate at distant places. In this study, a comprehensive study was conducted on the shifts in water quality, microalgal–bacterial dynamics, and temporal dynamics in red tilapia aquaculture ponds located in Guangzhou, Guangdong Province. A series of water samples were collected from the red tilapia aquaculture ponds across the whole culture period, and water quality indicators were measured. Additionally, the microalgal–bacterial dynamics were analyzed by high-throughput sequencing of 16S and 18S rRNA genes to (1) determine the shifts in microalgal–bacterial composition and diversities and (2) assess environmental drivers of these variations. The integration of water quality monitoring with microbiological sequencing transcends traditional silos of environmental science, offering a multidimensional view of aquatic health. By bridging chemical and biological perspectives, this approach offers unique scientific and practical advantages that transcend the capabilities of either conventional method alone.

## 2. Materials and Methods

### 2.1. Farming Management

**Figure 1:**
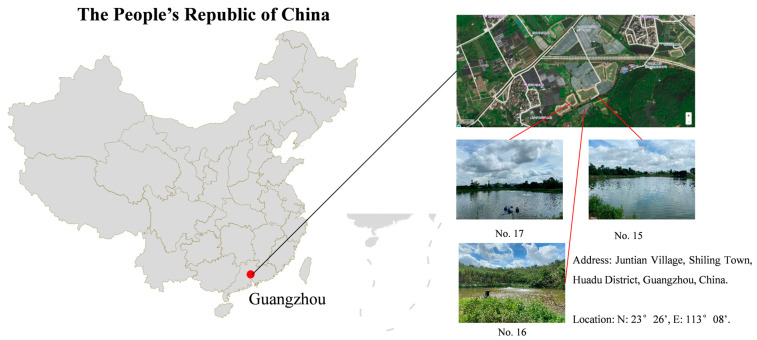
The diagram map of the study area and setting of sampling points.

The typical aquaculture ponds were located in Runyuan Aquatic Breeding Farm, Guangzhou City, Guangdong Province (Figure 1; 23°26′ N, 113°08′ E). Three ponds were named No. 15, No. 16, and No. 17, with areas of 11,333 m^2^, 3333 m^2^, and 4667 m^2^, respectively, and a water depth of 1.0 m during the culture period. The three experimental units (No. 15, 16, and 17) were selected according to several specific criteria. These included the depth of the ponds, their water quality, and their previous average production performance. The chosen ponds were of similar depth, water supply, and management methods to ensure consistency in the study. Water quality parameters, such as pH and dissolved oxygen, were also considered to be within acceptable ranges for red tilapia culture. Additionally, the previous average production performances of these ponds were evaluated to ensure they were representative of typical farming levels in the region. Underground water, pre-aerated and fertilized, was added into each pond before red tilapia stocking. The water was fertilized with urea (CH_4_N_2_O) at 0.5 mg/L upon initial pond filling to promote phytoplankton growth as supplementary natural feed. This aligns with local tilapia farming practices to enhance primary productivity. The red tilapia juveniles (~3 g) were transferred from nursery ponds into the three aquaculture ponds. These red tilapia juveniles were stocked at a density of 60 tails/m^3^, as recommended by prior farming practices in the region. During the culture period, the fish were fed a commercial feed with 4% of body weight at 9:00 and 17:00 daily. Feeding was adjusted biweekly based on biomass. The commercial feed of Luobeihou (≥30% crude protein, ≥5% crude lipid, ≤13% crude ash, ≤10% moisture) was purchased from Hailong Sci. & Tech. Co., Ltd. (Zhuhai, China). The culture period was from 6 July 2023 to 13 November 2023, totaling 130 days. Following the completion of the experiment, fish were caught and sold, resulting in a final body weight of approximately 500 g. The rainfall amounts during the culture period were 466.8 mm in July, 365.2 mm in August, 594.5 mm in September, 13.7 mm in October, 38.1 mm in November, and 9.1 mm in December.

### 2.2. Water Sampling and Characterization

During the culture period, water samples were respectively collected from three ponds (No. 15, No. 16, and No. 17) at an average of about once every 20 days. The accurate sample times in 2023 were as follows: Jul 06 (T1), Jul 29 (T2), Aug 14 (T3), Sep 05 (T4), Sep 25 (T5), and Nov 13 (T6). The T denotes the time-point of sampling (e.g., T1 = Time-point 1). All samplings were performed at 9:00 a.m. to minimize variation. Sampling was conducted during stable weather conditions (no precipitation 48 h prior) to minimize transient meteorological interference. Pond water was collected at 0.5 m below the water surface at each sampling point using a 5 L water collector. Water samples of 500 mL with three replicates were obtained from the middle layer of the water collector, stored in chilling containers, and transferred to the laboratory. Meanwhile, temperature, pH, dissolved oxygen, and salinity were detected by a YSI ProPlus multiparameter water quality instrument (YSI, Yellow Springs, OH, USA). In the laboratory, water samples were filtered through 0.45 μm pore-size sterile syringe filters (Pall Acrodisc^®^) for the analyses of six water quality indicators, including NH_4_^+^-N, NO_2_^−^-N, phosphate, sulfide, total chromium, and copper concentrations. The NH_4_^+^-N, NO_2_^−^-N, sulfide, total chromium, and copper concentrations of the water samples were measured by Nessler’s reagent (HJ 535-2009 [31]), N-(1-naphthyl) ethylenediamine (GB 7493-87 [32]), methylene blue (HJ 1226-2021 [33]), diphenyl carbamide (GB 7466-87 [34]), and sodium diethyldithiocarbamate (HJ 485-2009 [35]) spectrophotometric methods, respectively. Determination of phosphate was implemented with ion chromatography (HJ 669-2013 [36]). These measurement methods complied with the latest national and industry standards in China.

### 2.3. DNA Extraction and High-Throughput Sequencing

At each pond, four independent 500 mL water samples were collected and homogenized into a single composite sample (total 2000 mL per pond per time-point). From this composite, a 100 mL subsample with three replicates was filtered for DNA extraction, since a 100 mL volume provided sufficient biomass for high-yield DNA extraction while avoiding filter clogging. For DNA extraction, water samples were prefiltered through 200 μm mesh filters to remove large particles, and then 100 mL aliquots of each water sample were filtered through 0.22 μm microporous filter membranes (Millipore, Darmstadt, Germany). The total DNA was extracted from the filters using a DNeasy PowerSoil^®^ Kit (QIAGEN, Hilden, Germany) according to the manufacturer’s protocols. The extracted DNA samples were diluted and amplified in the full-length regions of the 16S and 18S rRNA genes. For 16S rRNA, the universal primer pairs 27F (ACTCCTACGGGAGGCAGCA) and 1492R (GGACTACHVGGGTWTCTAAT) were chosen [26]. For 18S rRNA, the universal primer pairs Euk-A (18S-F: CCAGCASCYGCGGTAATTCC) and Euk-B (18S-R: GATCCTTCTGCAGGTTCACCTAC) were chosen [37]. The PCR products were evaluated for integrity using 1.5% agarose (AG0100, Beijing LABLEAD Inc., Beijing, China) gel electrophoresis and quantified for concentration and purity using a microspectrophotometer. Samples that met the quality criteria were pooled for high-throughput sequencing on the Illumina NovaSeq platform (Biomarker Technologies Corporation, Beijing, China). The operational taxonomic units (OTUs) were clustered from the effective circular consensus sequencing reads at the 97% similarity level using USEARCH v.10.0 software and then filtered with a 0.005% threshold. After annotation, the community composition of each sample was calculated at various classification levels using QIIME2 software. Alpha and Beta diversities analysis was used to analyze changes in species composition during the culture period. Species diversity matrices were presented based on binary Jaccard. Principal component PCA (analysis), principal coordinate analysis (PCoA), nonmetric multidimensional scaling (NMDS), and partial least squares discriminant analysis (PLS-DA) were performed on the BMKCloud platform.

### 2.4. Statistical Analysis

Before analysis, the data were tested for normal distribution and variance homogeneity using the Shapiro–Wilk test and Hartley’s test, respectively. The one-way analysis of variance (ANOVA) was performed on inter-group differences using Duncan’s multiple comparisons. All statistical analyses were performed using the SPSS 26.0 (SPSS Inc., Chicago, IL, USA) software package. All results were expressed as the mean ± standard error of mean (SEM). The levels of significant differences were set to *p* < 0.05. Correlation analysis and visualization were performed on a website (https://www.chiplot.online, 31 October 2024).

## 3. Results

### 3.1. Water Quality Indicators and Correlation

**Figure 2:**
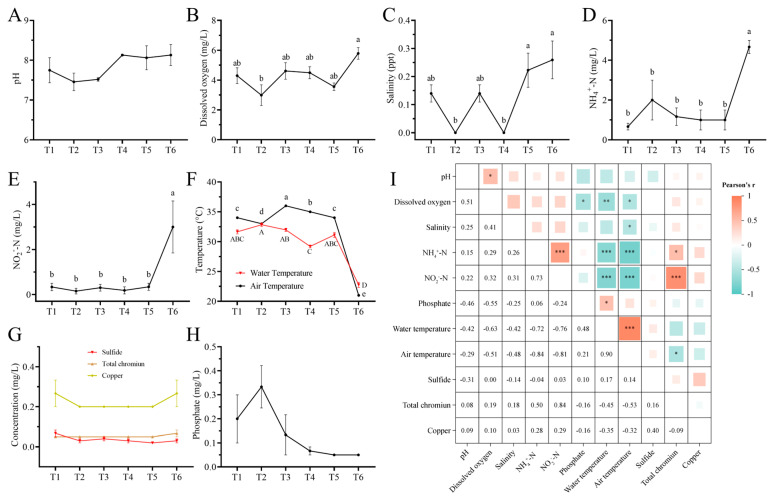
Water quality indicators at different culture stages and their correlations. Six accurate sample times in 2023 were Jul 06 (T1), Jul 29 (T2), Aug 14 (T3), Sep 05 (T4), Sep 25 (T5), and Nov 13 (T6), respectively. *n* = 3. Changes in (**A**) pH, (**B**) dissolved oxygen concentration, (**C**) salinity, (**D**) NH_4_^+^-N concentration, (**E**) NO_2_^−^-N concentration, (**F**) water and air temperatures, (**G**) sulfide, total chromium and copper concentrations, and (**H**) phosphate concentration across different time points or stages (T1–T6). Different letters indicate statistically significant differences (*p* < 0.05). Specifically, uppercase letters represent differences among water temperature treatments, and lowercase letters represent differences among air temperature treatments. (**I**) Correlation heat map showing Pearson’s r values between different water quality indicators; * *p* < 0.05, ** *p* < 0.01, *** *p* < 0.001.

Figure 2 shows the variations in water quality parameters throughout the culture period. The pH of the water ranged from 7.46 to 8.13 without significant variations, indicating weak alkalinity (Figure 2A). Dissolved oxygen concentration (Figure 2B) and salinity (Figure 2C) displayed fluctuations throughout the whole process. A significantly increased dissolved oxygen concentration at the end of the culture period (T6) was observed in comparison to T2, while the salinity in T5 and T6 was significantly higher than that in T2 and T3. The concentrations of NH_4_^+^-N (Figure 2D) and NO_2_^−^-N (Figure 2E) remained generally stable from T1 to T5, and a dramatic increase was documented in T6. On the contrary, sharply decreased water and air temperature were recorded in T6 (Figure 2G). There were no statistical significances for phosphate, sulfide, total chromium, and copper concentrations across the whole culture period (Figure 2F,H). As presented in Figure 2I, the correlation heat map showed that the dissolved oxygen concentration had significant negative correlations with the phosphate concentration (r = −0.55), water (r = −0.63), and air (r = −0.51) temperature. Furthermore, NH_4_^+^-N (r = −0.72 and −0.84) and NO_2_^−^-N (r = −0.76 and −0.81) concentrations showed a negative relationship with water and air temperature, while air temperature was negatively correlated with salinity (r = −0.48) and total chromium (r = −0.53) concentration. Conversely, the NH_4_^+^-N concentration exhibited positive relationships with NO_2_^−^-N (r = 0.73) and total chromium (r = 0.50) concentrations. Moreover, there were positive correlations between pH and dissolved oxygen concentration (r = 0.51), NO_2_^−^-N and total chromium (r = 0.84), and water temperature and phosphate concentration (r = 0.48), as well as air temperature (r = 0.90).

### 3.2. Bacterial Community Dynamics

**Figure 3:**
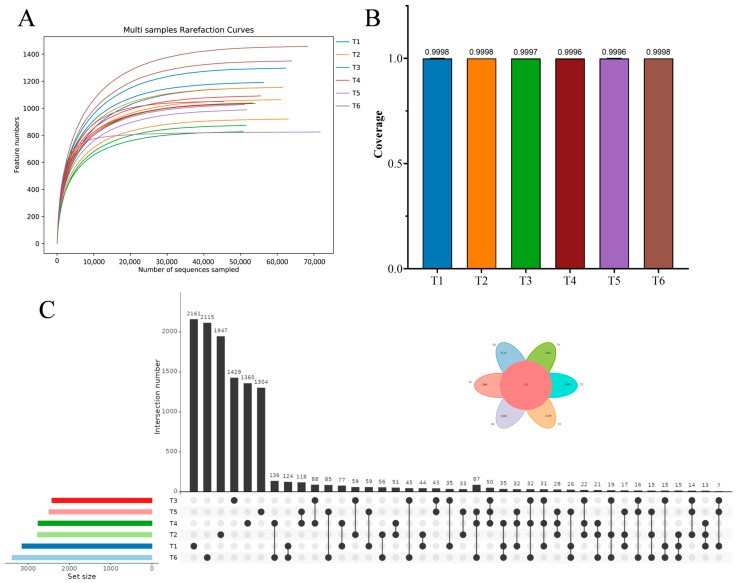
The rarefied curves (**A**), good’s coverage (**B**), and upset diagram (**C**) of water bacteria at different culture stages. Six accurate sample times in 2023 were Jul 06 (T1), Jul 29 (T2), Aug 14 (T3), Sep 05 (T4), Sep 25 (T5), and Nov 13 (T6), respectively. *n* = 3. (**A**) The plateau of rarefied curves indicates that the sequencing depth is sufficient to capture the majority of bacterial diversity in each sample. (**B**) All samples have near complete coverage (>0.99), confirming that the sequencing depth adequately represents the bacterial communities in each sample. (**C**) This upset diagram presented 2161, 1947, 1429, 1360, 1304, and 2115 unique operational taxonomic units (OTUs) at different culture stages, with 151 shared OTUs. The 151 shared OTUs refer to bacterial taxa that were consistently present across the whole culture period in the pond ecosystems.

Bacterial community dynamics across the whole culture period were assessed based on 16S rRNA sequencing. The rarefied curves for the observed species number tended to approach the saturation plateau (Figure 3A), and the Good’s coverage values exceeded 0.9996 (Figure 3B). These results indicated that sequencing depth and data output were adequate, and bacterial species in the water samples from the red tilapia ponds were almost identified in the libraries. The upset diagram presented 2161, 1947, 1429, 1360, 1304, and 2115 unique operational taxonomic units (OTUs) at different culture stages, with 151 shared OTUs (Figure 3C).

**Figure 4:**
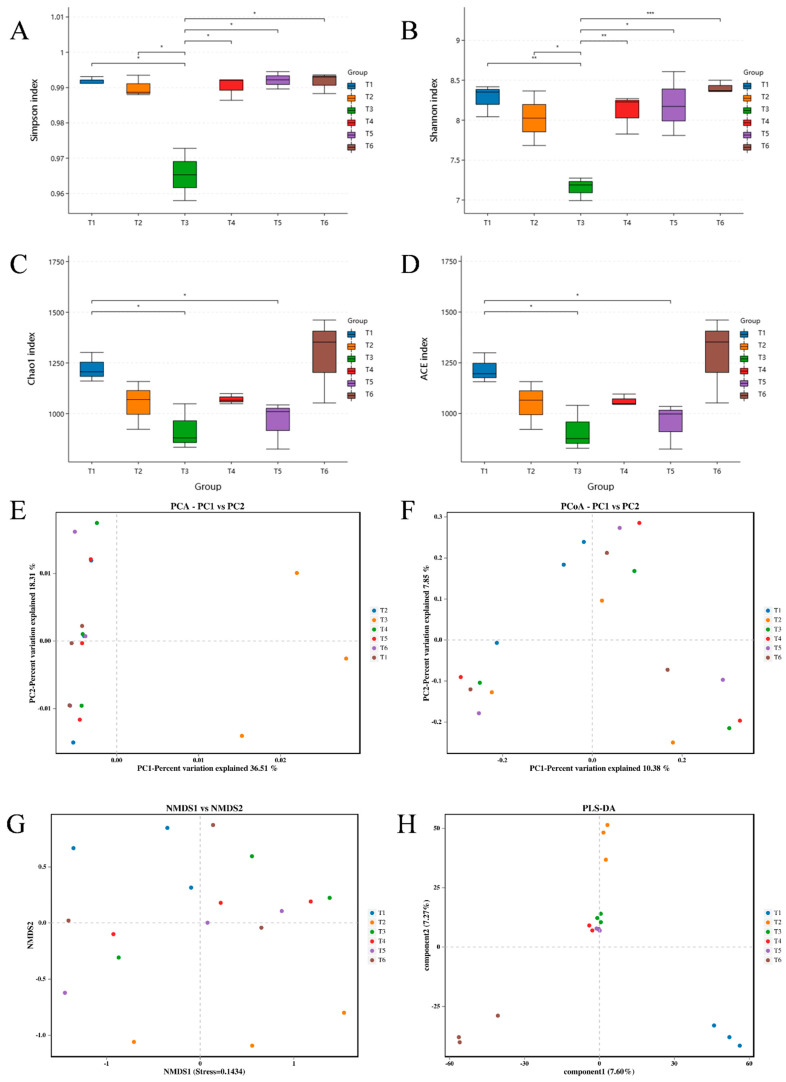
The α diversity and β diversity of water bacteria at different culture stages. Six accurate sample times in 2023 were Jul 06 (T1), Jul 29 (T2), Aug 14 (T3), Sep 05 (T4), Sep 25 (T5), and Nov 13 (T6), respectively. *n* = 3. (**A**) Simpson index. (**B**) Shannon index. (**C**) Chao 1 index. (**D**) ACE index. (**E**) PCA. (**F**) PCoA. (**G**) NMDS. (**H**) PLS-DA. Specifically, * indicates *p* < 0.05, ** indicates *p* < 0.01, and *** indicates *p* < 0.001.

Four α diversity indices (Simpson, Shannon, Chao 1, and ACE) were used to evaluate the shifts in bacterial diversity and richness at different culture stages. Significantly decreased bacterial diversity (Simpson and Shannon indices) was documented at the T3 stage in comparison with other stages (T1, T2, T4, T5, and T6) (Figure 4A,B). The bacterial richness (Chao 1 and ACE indices) showed a declining trend with increasing culture time, then increased sharply at the T6 stage (Figure 4C,D). The highest values of Simpson, Shannon, Chao 1, and ACE indices were recorded at the T6 stage. Furthermore, T3 was far from other stages (T1, T2, T4, T5, and T6) in the principal components analysis (PCA) plot (Figure 4E), while T1 and T2 were separated from other stages (T3, T4, T5, and T6) in the non-metric multidimensional scaling (NMDS) plot and from each other (Figure 4G). The principal coordinates analysis (PCoA) plot (Figure 4F) displayed a chaotic distribution across the whole culture period. Interestingly, T3, T4, and T5 were relatively close to each other, based on partial least squares discriminant analysis (PLS-DA, Figure 4H), implying a similar bacterial community. The PLS-DA plot also revealed separate clusters at the T1, T2, and T6 stages (Figure 4H).

**Figure 5:**
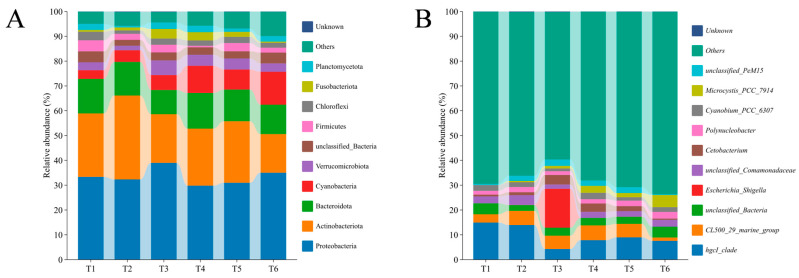
Shift in relative abundance of water bacterial composition at the phyla (**A**) and genera (**B**) taxonomic levels. Six accurate sample times in 2023 were Jul 06 (T1), Jul 29 (T2), Aug 14 (T3), Sep 05 (T4), Sep 25 (T5), and Nov 13 (T6), respectively. *n* = 3.

The top 10 most abundant phyla were Proteobacteria, Actinobacteriota, Bacteroidota, Cyanobacteria, Verrucomicrobiota, unclassified_Bacteria, Firmicutes, Chloroflexi, Fusobacteriota, and Planctomycetota, among which Proteobacteria was the most predominant phylum (Figure 5A). The relative abundances of Proteobacteria, Actinobacteriota, and Bacteroidota remained generally stable, except for a moderately increased Actinobacteriota at the T2 stage. The Cyanobacteria abundance was 3.51% in the initial phase of cultivation (T1). As the culture progressed, the relative abundance of this phylum in all water samples gradually increased, ultimately reaching 12.98% (T6). Conversely, Firmicutes showed a decreased trend (from 4.40% to 1.88%) with increasing culture time. Furthermore, the top 10 genera included *hgcI_clade*, *CL500_29_marine_group*, *unclassified_Bacteria*, *unclassified_Comamonadaceae*, *Escherichia_Shigella*, *Cetobacterium*, *Cyanobium_PCC_6307*, *Polynucleobacter*, *Microcystis_PCC_7914*, and *unclassified_PeM15* (Figure 5B). The relative abundance of *hgcI_clade* (from 14.91% to 7.18%) generally decreased with increasing culture time, while a contrary phenomenon was observed in terms of *Microcystis_PCC_7914* (from 0.02% to 4.31%). The relative abundances of *CL500_29_marine_group*, *Cetobacterium*, and *unclassified_PeM15* presented increasing trends from T1 to T3, then decreased from T3 to T6. It is worth noting that *Escherichia_Shigella* abundance accounted for over 15.63% of the bacterial sequences at the T3 stage, which was far richer than other stages (T1, T2, T4, T5, and T6).

### 3.3. Microalgal Community Dynamics

**Figure 6:**
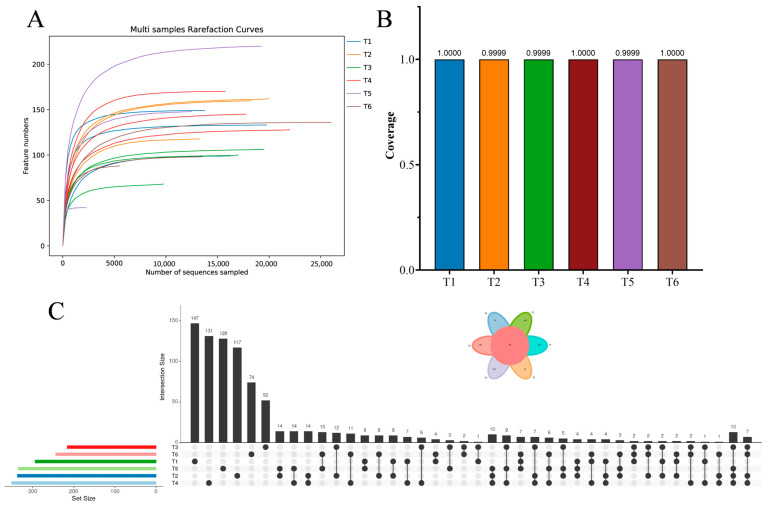
The rarefied curves (**A**), Good’s coverage (**B**), and upset diagram (**C**) of water microalgae at different culture stages. Six accurate sample times in 2023 were Jul 06 (T1), Jul 29 (T2), Aug 14 (T3), Sep 05 (T4), Sep 25 (T5), and Nov 13 (T6), respectively. *n* = 3. (**A**) The plateau of rarefied curves indicates that the sequencing depth is sufficient to capture the majority of microalgal diversity in each sample. (**B**) All samples have near complete coverage (>0.99), confirming that the sequencing depth adequately represents the microalgal communities in each sample. (**C**) This upset diagram presented 147, 117, 52, 131, 128, and 74 unique OTUs at different culture stages, with 44 shared OTUs. The 44 shared OTUs refer to microalgal taxa that were consistently present across the whole culture period in the pond ecosystems.

Microalgal community dynamics across the whole culture period were assessed based on 18S rRNA sequencing. The rarefied curves for the observed species number tended to approach the saturation plateau (Figure 6A), and the Good’s coverage values exceeded 0.9999 (Figure 6B). These results indicated that sequencing depth and data output were adequate, and microalgal species in the water samples from the red tilapia ponds were almost identified in the libraries. The upset diagram presented 147, 117, 52, 131, 128, and 74 unique OTUs at different culture stages, with 44 shared OTUs (Figure 6C).

**Figure 7:**
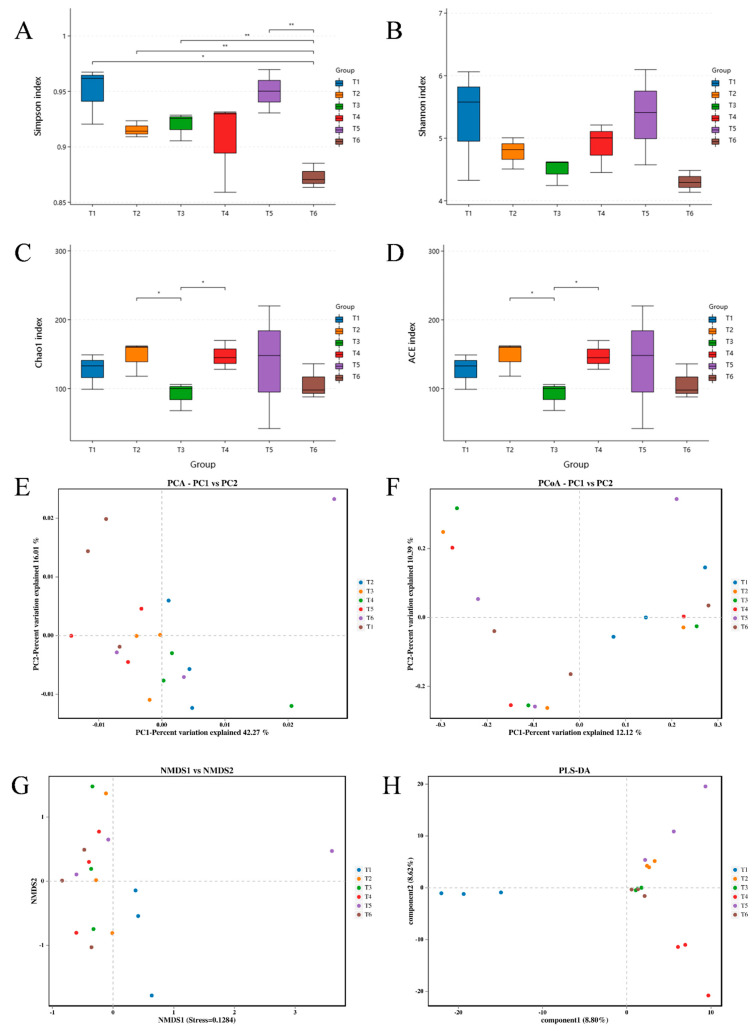
α diversity and β diversity of water microalgae at different culture stages. Six accurate sample times in 2023 were Jul 06 (T1), Jul 29 (T2), Aug 14 (T3), Sep 05 (T4), Sep 25 (T5), and Nov 13 (T6), respectively. *n* = 3. (**A**) Simpson index. (**B**) Shannon index. (**C**) Chao 1 index. (**D**) ACE index. (**E**) PCA. (**F**) PCoA. (**G**) NMDS. (**H**) PLS-DA. Specifically, * indicates *p* < 0.05, and ** indicates *p* < 0.01.

Four α diversity indices (Simpson, Shannon, Chao 1, and ACE) were used to evaluate the shift in microalgal diversity and richness at different culture stages. The microalgal diversity (Simpson and Shannon indices) gradually decreased with increasing culture time (except T5), and statistically significant decline was documented in the Simpson index at the T6 stage when compared to other stages (T1, T2, T3, and T5) (Figure 7A,B). The microalgal richness (Chao 1 and ACE indices) showed a slight fluctuation across the whole culture period (Figure 7C,D). Further statistical tests revealed that Chao 1 and ACE indices at the T3 stage were significantly lower than those at the T2 and T4 stages. Furthermore, PCA (Figure 7E), PCoA (Figure 7F), and NMDS (Figure 7G) plots displayed chaotic distributions throughout the culture period. Interestingly, T2 and T5 were relatively close to each other based on PLS-DA, as well as T3 and T6 (Figure 7H), implying a similar microalgal community. The PLS-DA plot also revealed separate clusters at the T1 and T4 stages (Figure 7H).

**Figure 8:**
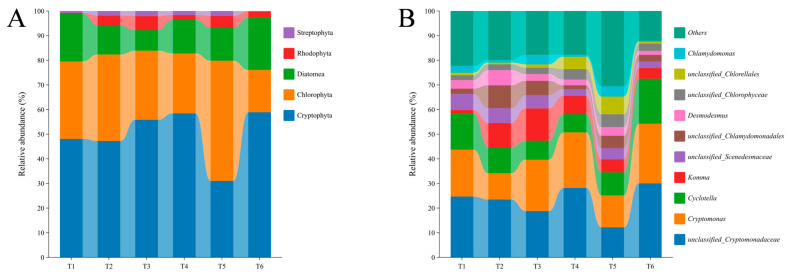
Shifts in the relative abundance of water microalgal composition at phyla (**A**) and genera (**B**) taxonomic levels. Six accurate sample times in 2023 were Jul 06 (T1), Jul 29 (T2), Aug 14 (T3), Sep 05 (T4), Sep 25 (T5), and Nov 13 (T6), respectively. *n* = 3.

A total of five microalgal phyla, including Cryptophyta, Chlorophyta, Diatomea, Rhodophyta, and Streptophyta, were identified from the 18S rRNA sequencing of all water samples (Figure 8A). The Cryptophyta abundance gradually increased from 48.81% to 62.73% with increasing culture time (except T5 with 29.22%), while a gradually decreased trend (from 32.42% to 19.43%) was recorded in terms of Chlorophyta abundance (except T5 with 48.88%). The relative abundance of Diatomea showed a declining trend from T1 (17.78%) to T3 (9.55%), then rose from T3 to T6 (15.41%), whereas the changes in Rhodophyta and Streptophyta abundances demonstrated the opposite phenomenon with increasing culture time. Furthermore, the top 10 genera included *unclassified_Cryptomonadaceae*, *Cryptomonas*, *Cyclotella*, *Komma*, *unclassified_Scenedesmaceae*, *unclassified_Chlamydomonadales*, *Desmodesmus*, *unclassified_Chlorophyceae*, *unclassified_Selenastraceae*, and *unclassified_Chlorellales*, among which *unclassified_Cryptomonadaceae*, *Cryptomonas*, *Cyclotella*, and *Komma* were the most predominant genera (Figure 8B). The relative abundances of *unclassified_Cryptomonadaceae* (ranging in abundance from 9.79% to 31.95%), *Cryptomonas* (ranging in abundance from 10.13% to 26.11%), and *Cyclotella* (ranging in abundance from 6.01% to 13.31%) remained generally stable, although there were some fluctuations throughout the culture period. The *Komma* abundance presented an increasing trend from T1 (1.44%) to T3 (13.90%), then decreased from T3 to T6 (4.21%).

### 3.4. Correlation Analysis Between Water Quality Indicators and Microalgal–Bacterial Communities

**Figure 9:**
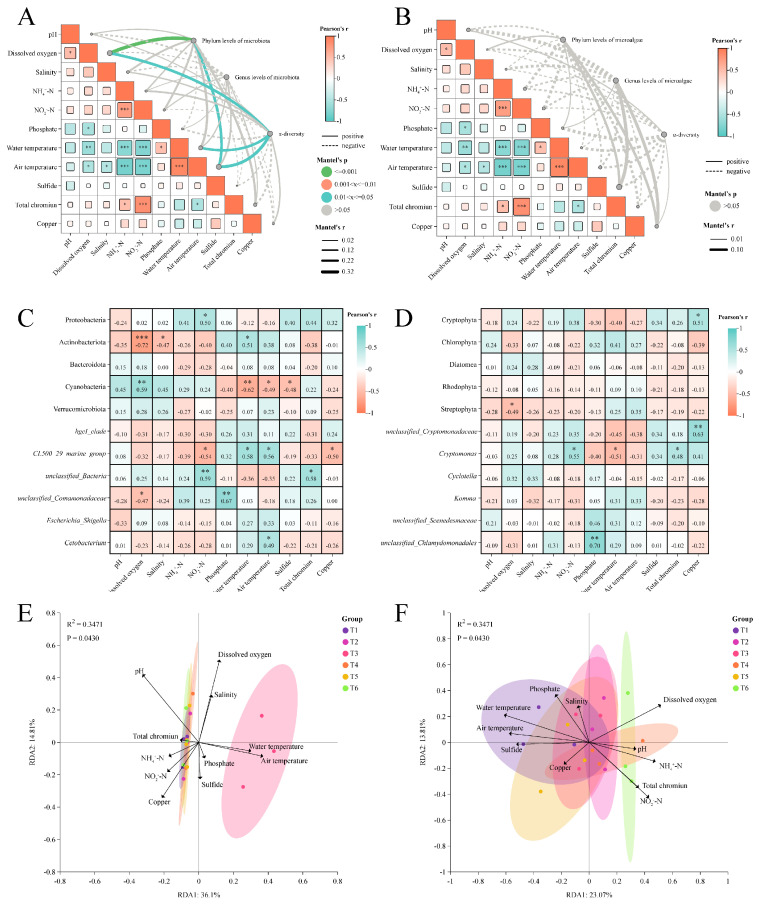
Correlation analysis between water quality indicators and microalgal–bacterial communities. Six accurate sample times in 2023 were Jul 06 (T1), Jul 29 (T2), Aug 14 (T3), Sep 05 (T4), Sep 25 (T5), and Nov 13 (T6), respectively. *n* = 3. Mantel test between environmental variables and water microbiota, including bacteria (**A**) and microalgae (**B**). Pearson correlation analysis between environmental variables and the top five phyla and genera in bacterial (**C**) and microalgal (**D**) communities. RDA revealed the major environmental variables in bacterial (**E**) and microalgal (**F**) dynamics. Specifically, * indicates *p* < 0.05, ** indicates *p* < 0.01, and *** indicates *p* < 0.001.

In the present study, the correlations between all water quality indicators and the evolutions in phyla, genera, and α diversities of microalgal–bacterial communities were analyzed by the Mantel test (Figure 9A,B). The bacterial phyla were significantly correlated with dissolved oxygen and air temperature, while α diversities of the bacterial community were significantly correlated with dissolved oxygen, water, and air temperature (Figure 9A). Water quality indicators were certainly linked to the evolutions in phyla, genera, and α diversities of the microalgal community, although no statistical significance was shown by the Mantel test (Figure 9B). As shown in Figure 9C, the dissolved oxygen concentration was negatively correlated with Actinobacteriota and *unclassified_Comamonadaceae* but positively correlated with Cyanobacteria. The concentration of NO_2_^−^-N was negatively correlated with *CL500_29_marine_group* but positively correlated with Proteobacteria and *unclassified_Bacteria*. Moreover, the water and air temperature were strongly correlated with the most dominant bacteria (Figure 9C). In terms of microalgae, there were positive correlations between *Cryptomonas* and the NO_2_^−^-N concentration, as well as *unclassified_Scenedesmaceae* and the phosphate concentration (Figure 9D). The negative correlations were observed between Streptophyta and the dissolved oxygen concentration, as well as *Cryptomonas* and water temperature (Figure 9D). The RDA plots display the impacts of water quality indicators on the microalgal–bacterial communities throughout the culture period (Figure 9E,F). Dissolved oxygen concentration, water, and air temperature were main factors that were positively correlated with the bacterial community at the T3 stage but had negative correlations at other stages. The pH was most positively associated with the water bacteria at the T1, T2, T4, T5, and T6 stages, followed by copper, NH_4_^+^-N, and NO_2_^−^-N concentrations, while negative correlations between the four water quality indicators and the bacterial community were observed at the T3 stage. In addition, dissolved oxygen, NH_4_^+^-N, and NO_2_^−^-N concentrations were main factors that were positively correlated with the microalgal community across the culture period, especially T6. It was also recorded that water and air temperature exhibited strongly negative associations with the microalgal community.

## 4. Discussion

Future aquaculture aims to achieve sustainable development, including sustainable aquaculture growth and environmental conservation [1]. A critical precondition for all of this is maintaining good water quality and possessing superior buffering capacity against a dynamic external environment [38,39,40]. Water quality is usually analyzed by measuring many indicators, such as pH, temperature, dissolved oxygen, NH_4_^+^-N, NO_2_^−^-N, phosphate, sulfide, and total chromium, as well as copper concentration. Copper is an essential metal utilized in aquaculture activities for both nutritional and disease control. Conversely, copper can present a risk of exposure to biota, depending on the overconcentration. For example, 10.0 mg copper/kg body weight could impair sperm quality, antioxidant response, and reproduction in Nile tilapia (*O. niloticus*) [41]. Similarly, chromium is considered to be one of the vibrant metals because of its cumulative deleterious effects on living organisms [42]. It has been reported that that chromium (3.2 mg/L) is highly toxic to striped catfish (*Pangasianodon hypophthalmus*), inducing nuclear and cellular erythrocyte alterations and damage to the gills, liver, kidney, and genomic DNA [43]. Therefore, it is necessary to detect heavy metal levels in sustainable aquaculture. In the present study, total chromium and copper concentrations across the whole culture period were low, stable, and non-lethal for red tilapia. In aquaculture, the most significant factors affecting water quality are fish feces and residual feeds, which are decomposed by microorganisms into inorganic small molecules such as NH_4_^+^-N, leading to the enhancement of free nitrogen concentration in water [44]. NO_2_^−^-N is an intermediate product of the conversion of NH_4_^+^-N to NO_3_^−^-N [45]. High concentrations of NH_4_^+^-N and NO_2_^−^-N are toxic to fish and might cause excessive proliferation of pathogenic microalgae–bacteria [38,44,46]. In the present study, the water quality indicators of red tilapia aquaculture ponds changed with increasing culture time. At the end of the culture period (T6, 13 November 2023), NH_4_^+^-N and NO_2_^−^-N concentrations were sharply increased, whereas air and water temperature expressed significant reductions. A possible explanation for this might be that the continued accumulation of fish feces and residual feeds in water led to increased NH_4_^+^-N and NO_2_^−^-N concentrations. In addition, the season transfer-induced temperature decrease could suppress feed intake and digestion, which magnified the accumulation. Future studies should include measurements such as total organic carbon, chemical oxygen demand, or suspended solids to better characterize organic matter dynamics and their ecological impacts. The speculation was partially supported by further analysis that NH_4_^+^-N and NO_2_^−^-N concentrations showed a strongly negative relationship with water and air temperature, while the NH_4_^+^-N concentration exhibited positive relationships with NO_2_^−^-N and total chromium concentrations. These results indicated that NH_4_^+^-N and NO_2_^−^-N concentrations should be promptly monitored at the end of the culture period, especially with drastic temperature changes. Moreover, regular monitoring of NH_4_^+^-N and NO_2_^−^-N concentrations is important in feed-driven aquaculture, particularly during the stages (approximately three months after the beginning of feeding) when organic waste accumulation tends to increase. In line with this study, there is a consistent increase in total ammonia nitrogen, nitrite, and nitrate concentrations throughout the culture period of Nile tilapia (*O. niloticus*) [47]. Artificial interventions, such as changing the water and supplementing with nitrifying bacteria, will be considered to ensure that NH_4_^+^-N and NO_2_^−^-N concentrations can be maintained at a low level, thereby preventing and controlling the aquaculture risks. The changes in environmental factors will drive the dynamic shift in microbial communities in the aquaculture water, and the changes in microbial communities will, in turn, regulate the aquaculture environment, thereby affecting the aquaculture ecosystem. Microorganisms play a vital role in linking environmental factors and ecosystems [48,49]. Therefore, we will further analyze the microalgal–bacterial dynamics and their correlation with water quality indicators.

The microbial community depends on the interaction between microorganisms and environmental factors, which is the basis for their ecological functions [48,50,51]. Moreover, the diversity index is an important parameter to measure the diversity and richness in a specific region or ecosystem community [52]. In the present study, bacterial diversity decreased until the T3 stage (14 August 2023) and then increased. In addition, Proteobacteria, Actinobacteriota, and Bacteroidota, as the predominant phyla, remained generally stable in red tilapia aquaculture ponds. In accordance with the present results, previous studies demonstrated that the dominant bacterial phyla were Cyanobacteria, Proteobacteria, Actinobacteriota, and Bacteroidota in the pond water of tilapia (*O. niloticus*) [53,54] and grass carp (*Ctenopharyngodon idella*) [55]. Moreover, *hgcI_clade* and *CL500-29_marine_group* were commonly considered dominant species in freshwater ecosystems that showed superior competitiveness and proliferative capacity in oligotrophic freshwater [56,57,58]. Interestingly, the relative abundance of *hgcI_clade* and *CL500-29_marine_group* in aquaculture water generally decreased with increasing culture time, indicating the enhancement of nutrient concentrations. In feed-driven aquaculture systems, the organic matter in the water continued to increase with increasing culture time. The nutrient-rich conditions were detrimental to the growth of *hgcI_clade* and *CL500-29_marine_group*, eventually decreasing *hgcI_clade* and *CL500-29_marine_group* abundances. This finding may be due to the continued accumulation of fish feces and residual feeds. Additionally, *hgcI_clade* and *CL500-29_marine_group* were positively correlated with temperature and phosphate and negatively correlated with dissolved oxygen, NH_4_^+^-N, and NO_2_^−^-N concentrations. In the present study, pH, dissolved oxygen concentration, and temperature were main factors in the bacterial dynamic. It has been also reported that the *CL500-29_marine_group* can effectively use different forms of carbon-based compounds, and the *hgcI_clade* has a strong ability to use carbon-containing compounds and can dissolve organic carbon at low concentrations in low-temperature water [59]. Among the most prevalent bloom-forming cyanobacteria is the genus *Microcystis* [60], which can lead to ecosystem disruption through shading [61] and oxygen depletion [62] and represents a public health threat via the production of the hepatotoxin and microcystin [63]. The relative abundance of *Microcystis_PCC_7914* (from 0.02% to 4.31%) generally increased with increasing culture time, indicating accumulated nutrients. Farmers need to pay attention to the occurrence of *Microcystis* blooms during the stages (approximately three months after the beginning of feeding) when organic waste accumulation tends to increase. The high relative abundance of *Escherichia_Shigella* observed in the mid-culture stage (T3) may reflect an increase in organic matter and nutrient input, potentially linked to accumulated feed residues and metabolic waste from the cultured organisms. These results indicated that the bacterial community is seriously impacted by environmental factors and thereby might affect the health and welfare of red tilapia. Similarly, during the turbid-turned stage in small greenhouses, microorganisms, on the one hand, played the role of probiotics to improve the disease resistance of shrimp, such as Actinobacteriota. On the other hand, in the late turbid-turned stage, due to the changes in the water environment, bacterial–algal systems formed by heterotrophic bacteria, autotrophic bacteria, and algae were enriched in a directed manner, such as *Chlorella*, *Marivita,* and AOB. They converted ammonia nitrogen into nitrite and biomass, which is important for a healthy aquaculture environment in small greenhouses and enhancing growth performance [26].

The shape of an optimal aquaculture environment also requires a beneficial microalgal balance for hydrobios. The changes in the dominant species often determine the direction of microalgal balance, affecting the water environment and healthy development of aquaculture. For instance, several reports have shown that *Spirulina platensis* could alleviate hepatic injury and inflammation in catfish (*Clarias gariepinus*) and common carp (*Cyprinus carpio* L.), respectively, and prevent exposure to lead (Pb) [64] and atrazine [65]. These prior findings provided evidence that algae could modulate physiological and immunological functions across aquatic species, which may eventually affect aquaculture efficiency. Thus, it is significant for economically efficient and sustainable modern aquaculture to study the microalgal community in aquaculture water and the effects of environmental factors on the succession of microalgae communities. In this study, the Cryptophyta abundance gradually increased from 48.81% to 62.73% with increasing culture time (except T5), and a reverse trend was recorded in Chlorophyta abundance. *Komma*, belonging to the phylum Cryptophyta, prefers to grow in water rich in organic matter and nitrogen and is sensitive to ambient temperature [66]. The abundance of the predominant genus *Komma* presented an increasing trend from T1 to T3, then decreased from T3 to T6. These results indicated that the microalgal community exhibited dynamic changes at different culture stages. The initial rise of *Komma* abundance was primarily driven by organic matter and nitrogen accumulation, creating favorable nutrient conditions for this genus. Subsequently, the decline from T3 onward correlated strongly with the decreasing water temperature, which likely suppressed the metabolic activity and growth rates of *Komma*. These nutrient–temperature interactions highlight how culture stage transitions shift microalgal dynamics. In the mid-to-late culture period, nutrient accumulations in aquaculture water can lead to the rapid growth of Cyanobacteria while releasing cyanotoxins, eventually inducing microalgal dysbiosis and disease outbreak [67,68]. Interestingly, dissolved oxygen, NH_4_^+^-N, and NO_2_^−^-N concentrations were main factors that were positively correlated with the microalgal community across the culture period; meanwhile, it was also recorded that temperature exhibited strongly negative associations with the microalgal community. The present study again confirmed the significant influence of environmental factors in establishing dominant microalgal species [69,70,71]. A healthy microalgal community depends on timely and suitable regulation and management. In aquaculture, comprehensive measures should be taken for the regulation and management, since the water eco-environment is a dynamic balance involving diverse environmental factors. The interaction between environmental factors and the microalgal–bacterial community can potentially be harnessed, but its complexity requires further research. Given the potential influence of seasonal and/or weather effects, as well as rainfall events, on environmental monitoring data, weather parameters will be tracked more explicitly on a daily basis in future studies. This will be conducive to identifying environmental drivers. Monitoring the growth and biomass of aquatic organisms is a fundamental aspect in aquaculture, since these parameters are decisive for making decisions on cultivation strategies, species selection, and evaluation of the efficiency of production systems. It has been shown that growth and biomass not only reflect the biological success of the organisms, but also allow correlating water quality and environmental conditions with productive performance [26], thus optimizing the sustainability and profitability of the aquaculture system. The omission of these indicators limits the applicability of the results for the productive sector and makes comparisons with other works in the area difficult. These parameters should be systematically included in future studies to strengthen the relevance and impact of research in aquaculture practice.

## 5. Conclusions

In conclusion, this study revealed regime shift in the water quality and microalgal–bacterial community with increasing culture time in red tilapia aquaculture ponds. The NH_4_^+^-N and NO_2_^−^-N concentrations increased by 597% (0.67 ± 0.17 to 4.67 ± 0.33 mg/L) and 782% (0.34 ± 0.16 to 3.00 ± 1.15 mg/L), respectively, from T1 to T6, contrary to water and air temperature. The relative abundance of *hgcI_clade* and *CL500-29_marine_group* in aquaculture water generally decreased with the extension of aquaculture time. The abundance of *Komma* increased from T1 to T3 and decreased from T3 to T6. Furthermore, pH, dissolved oxygen, and temperature were the main factors in bacterial dynamics, while dissolved oxygen, NH_4_^+^-N, and NO_2_^−^-N concentrations were the main factors in microalgal dynamics.

## Data Availability

The original contributions presented in this study are included in the article. Further inquiries can be directed to the corresponding author.
